# 

**Published:** 1828-09

**Authors:** 


### MONTHLY LIST OF MEDICAL BOOKS.

[*Medical Works cannot be entered on this List except a copy be sent for the purpose ; the titles of Books having frequently been transmitted to us, as published, which have not appeared for weeks, or even months, after.*]

A Letter to the Right Hon. Robert Peel, Secretary of State, &c. &c. on some of the Impediments, Defects, and Abuses existing in the present System of Medical Education; with Suggestions for their Removal and Correction. By Henry William Dewhurst, Surgeon, Lecturer on Anatomy, &c. &c. —London. Stitched, pp. 51.

A Manual of the Anatomy, Physiology, and Diseases of the Eye and its Appendages. By S. J. Stratford, Member of the Royal College of Surgeons in London: Surgeon to the Dispensary for Diseases of the Eye; and late senior Assistant Surgeon of the 72d, or Duke of Albany's own Highlanders.—8vo. Longman and Co. London, 1828.

### NOTICES.

*The Communications of* Dr. Blake, Mr. Kitson, *and* Dr. Bow, *have been received.*

